# Cell Membrane-Camouflaged Nanocarriers for Cancer Diagnostic and Therapeutic

**DOI:** 10.3389/fphar.2020.00024

**Published:** 2020-02-04

**Authors:** Shengxian Li, Jianhua Liu, Mengyao Sun, Jixue Wang, Chunxi Wang, Yinghao Sun

**Affiliations:** ^1^ Department of Urology, the First Hospital of Jilin University, Changchun, China; ^2^ Department of Urology, Shanghai Changhai Hospital, Second Military Medical University, Shanghai, China

**Keywords:** biological membrane, nanoparticle, drug delivery, cancer treatment, imaging

## Abstract

Cell membrane (CM)-camouflaged nanocarriers (CMNPs) are the tools of a biomimetic strategy that has attracted significant attention. With a wide range of nanoparticle cores and CMs available, various creative CMNP designs have been studied for cancer diagnosis and therapy. The various functional CM molecules available allow CMNPs to demonstrate excellent properties such as prolonged circulation time, immune escape ability, reduced systemic toxicity, and homologous targeting ability when camouflaged with CMs derived from various types of natural cells including red and white blood cells, platelets, stem cells, and cancer cells. In this review, we summarize various CMNPs employed for cancer chemotherapy, immunotherapy, phototherapy, and *in vivo* imaging. We also predict future challenges and opportunities for fundamental and clinical studies.

## Introduction

Cancer is a worldwide health problem and is currently the most important hindrance to life expectancy improvement. In 2015, the World Health Organization (WHO) estimated that cancer is the first or second most common cause of death among people under age 70 in most countries (Bray et al., 2018). According to updated nationwide cancer statistics from the National Central Cancer Registry of China (NCCRC), there were 3.8 million new cancer cases (crude incidence rate: 278.07/100,000) and 2.3 million cancer deaths (crude incidence rate: 278.07/100,000) (Chen et al., 2018). Cancer cells exhibit uncontrollably rapid proliferation *via* growth signal self-sufficiency, resisting growth inhibitory, and escaping from apoptotic signals (Saez-Rodriguez et al., 2015). Cancer cells can escape from immune surveillance and metastasize from origin sites to other organs. They can even generate adaptive strategies in response to effective treatments *via* further gene mutation (Gatenby and Brown, 2018). Chemotherapy, radiotherapy, and immunotherapy are presently the main clinical cancer treatment methods. Other treatment strategies such as phototherapy and gene therapy are also being developed. However, these treatment strategies have demonstrated unsatisfactory results due to poor pharmacokinetics, low permeability, low targeting ability, and severe side-effects.

Due to the previously mentioned shortcomings of cancer treatment strategy, nanoparticle (NP)-based drug delivery systems (DDSs) have been widely studied for tumor diagnosis and treatment (Dan et al., 2007). Particles 1–1,000 nm in size are defined as nanoparticles and offer good drug delivery characteristics. Nanoparticles 10–100 nm in size are proven to offer the highest delivery efficacy (Narain et al., 2017). Various types of nanomaterials such as polymers, liposomes, and metals have been developed for the delivery of therapeutic agents for cancer treatment. The cooperation between payload and nanomaterial can produce effective drug delivery *via* passive or active targeting strategies and can offer high drug loading capacities, increased circulation times, and reduced systemic toxicities. In the passive approach, a nanoparticle-based DDS can deliver therapeutic agents effectively *via* the enhanced permeation and retention (EPR) effect. An active antitumor agent approach can be used when the nanomaterials are synthesized to exhibit environmentally responsive characteristics or targeting ligands. Meanwhile, the cooperation of varying payloads *via* nanoparticle-based DDS can elicit diversification effects, like the implementation of chemoimmunotherapy (Fang et al., 2018).

However, the foreign nature of nanoparticles makes it easy for the immune system to recognize and eliminate them easily. In order to achieve more efficient drug delivery with a low clearance rate, biomimetic nanoparticles have been designed to prolong circulation time and evade clearance by the immune system. PEGylation has been widely used to decrease nanoparticle elimination. However, it has been reported that anti-PEG antibodies can be produced after repeated administration of PEGylated nanoparticles, and this might promote the elimination these nanoparticles (Lubich et al., 2016). In contrast, lipids are a major part of the cell membrane (CM). They have been used to produce biomimetic liposomes in order to mimic biological membranes. However, these liposomes lack structural integrity and stability. This restricts their application as DDSs (Maurer et al., 2001).

**Scheme 1:**
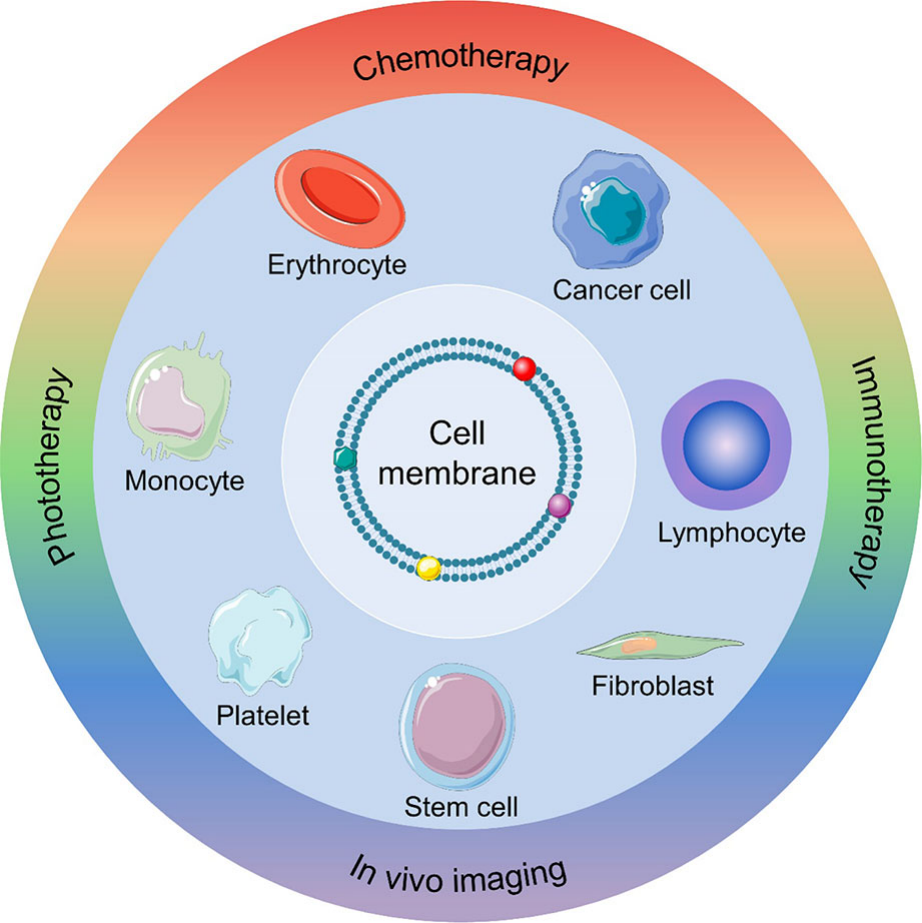
Schematic illustration of a nanocarrier-assisted cell membrane designed for cancer diagnosis and treatment.

A range of new biomimetic nanoparticle-based DDSs have recently been developed to combine the benefits of natural and synthetic nanomaterials (Kroll et al., 2017; Bose et al., 2018). CMs are natural-source ingredients that can be coated onto nanoparticles to produce CM-coated nanoparticles (CMNPs) with cell-like behaviors. The nanoparticle core can protect various therapeutic cargos *via* its high structural integrity and stability. In addition, CMs can provide CMNPs with prolonged circulation time, targeting ability, and other source cell properties. For instance, red blood cell (RBC) membrane could be used for immune evasion and prolonging circulation time. White blood cell (WBC) membranes could be employed to camouflage nanoparticle for evading opsonization and reticuloendothelial system (RES) clearance and targeting inflamed sites. Cancer cell membrane (CCM) could act as the tumor-targeting navigator and the source of tumor-associated antigens (TAAs). In **Table 1**, we summarize various CM-coated nanoparticles developed for cancer diagnosis and treatment. In this review, we provide an overview of CMNP-based DDSs as tumor diagnostic and therapeutic agents and discuss potential clinical applications (**Scheme 1**).

**Table 1:** The antitumor application of various CM-coated nanoparticles.

Therapeutic strategies	Membrane coat	Core nanoparticle	Tumor model	Reference
Chemotherapy	CCM derived from HepG2 cell	PLGA-DOX	HepG2 cell	(Xu et al., 2019)
	CCM derived from MCF-7 cell	PLGA-DOX and Hb	MCF-7 cell	(Tian et al., 2017)
	RBM	PLA-DOX	Kasumi-1 cell	(Aryal et al., 2013)
	Monocyte cell membrane	PLGA-DOX	MCF-7 breast cancer cell	(Krishnamurthy et al., 2016)
	Macrophage membrane	Cationic 2-aminoethyldiisopropyl group (PPiP)-functionalized PEGylated poly(β-amino ester)-PTX	MDA-MB-231 breast cancer cell	(Zhang et al., 2018)
	PM	Nanovehicle-DOX and tumor necrosis factor (TNF)‐related apoptosis inducing ligand (TRAIL)	MDA-MB-231 breast cancer cell	(Hu et al., 2015)
	Composite cell membrane (derived from leukocytes and HN12 tumor cell)	Liposomal nanoparticles-PTX	HN12 head and neck tumor cell and B16 melanoma cell	(He et al., 2018)
	4T1 cell- derived CCM	MSN-DOX and ICG	4T1 breast cancer cell	(Ding et al., 2019)
	RBM	MB and Pt loaded gelatin nanogel core (MPNGs)	4T1 breast cancer cell	(Zhai et al., 2018)
	RBM	Hollow mesoporous PB nanoparticles-DOX	4T1 breast cancer cell	(Chen et al., 2017a)
Immunotherapy	Composite cell membrane (derived from leukocytes and platelet)	IMBs	Blood samples of breast cancer patients	(Rao et al., 2018)
	Neutrophil membrane	PLGA nanoparticles-Carfilzomib	4T1 breast cancer cell	(Kang et al., 2017)
	CCM derived from B16-OVA cell	PLGA nanoparticle-R837	B16-OVA cancer cell	(Yang et al., 2018)
	CCM derived from RM-1 cell	PLGA nanoparticle-R837	RM-1 prostate cancer cell	(Li et al., 2019)
	RBM	BPQDs	4T1 breast cancer cell	(Liang et al., 2019)
	CCM derived from surgical 4T1 tumors	BPQDs	4T1 breast cancer cell	(Ye et al., 2019)
Photothermal therapy	Macrophage membrane	Fe_3_O_4_ nanoparticle	MCF-7 human breast cancer cell	(Meng et al., 2018)
	Composite cell membrane (derived from RBCs and MCF-7 cancer cell)	Melanin nanoparticle	MCF-7 human breast cancer cell	(Jiang et al., 2019)
	HA-decorated RBM	PB nanoparticle-CS-6	MDA-MB-231 cell	(Liu et al., 2019)
	Composite cell membrane (derived from RBCs and B16-F10 melanoma cell)	Hollow copper sulfide nanoparticles-DOX	B16-F10 melanoma cell	(Wang et al., 2018)
	RBM	Poly(caprolactone)‐ester endcap polymer (PCL) nanoparticle-PTX	4T1 breast cancer cell	(Su et al., 2016)
	RBM	Hollow mesoporous PB nanoparticles-DOX	4T1 breast cancer cell	(Chen et al., 2017a)
	Anti-EpCam antibody-modified RBM	Gold nanoparticle-PTX	4T1 breast cancer cell	(Zhu et al., 2018)
	RBM	BPQDs	4T1 breast cancer cell	(Liang et al., 2019)
Photodynamic therapy	RBM	NaYF_4_:Yb/Er UCNP	B16 melanoma cell	(Ding et al., 2015)
	PM	PLGA nanoparticle-verteporfin	4T1 breast cancer cell	(Xu et al., 2018)
	STM	β-NaYF4:Yb^3+^, Er^3+^ UCNP	HeLa human cervical cancer cell	(Gao et al., 2016)
	CCM derived from 4T1 cancer cell	C16-K(PpIX)RRKK-PEG-COOH	4T1 breast cancer cell	(Qiu et al., 2018)
	CCM derived from SGC7901 cell	CM/SLN/Ce6	SGC7901 cell	(Yang et al., 2019)
	RBM	Methoxypoly(ethylene glycol)-block-poly(D,L-lactide) (PEG-bPDLLA)-PTX and TPC	HeLa human cervical cancer cell	(Pei et al., 2018)
	CCM derived from 4T1 cancer cell	MOF-GOx and catalase	4T1 breast cancer cell	(Li et al., 2017)
	CCM derived from SMMC-7721 cell	Polyethyleneimine (PEI)-modified, styrene (St), and acrylic acid (AA)-crosslinked SPIO NP	SMMC-7721 cell	(Li et al., 2018a)
	Activated fibroblast	Poly-(cyclopentadithiophene-alt-benzothiadiazole) nanoparticle	4T1 breast cancer cell	(Li et al., 2018b)
*In vivo* imaging	RBM	Fe_3_O_4_ NP	–	(Rao et al., 2016b)
	STM	SPIO NP	TRAMP-C1 mouse prostate cancer cell	(Lai et al., 2015)
	RBM	^99m^Tc-labeled EMs	–	(Gangadaran et al., 2018)
	CCM derived from 4T1 cancer cell	^89^Zr-labeled multicompartment membrane-derived liposomes-tetrakis(4-carboxyphenyl) porphyrin	4T1 breast cancer cell	(Yu et al., 2018)
	CCM derived from MDA-MB-435 human breast cancer cell, DU 145 human prostate cancer cell, CAL 27 human squamous cancer cell, and HCT 116 human colorectal cancer cell	β-NaYF_4_:Er^3+^, Yb^3+^ UCNP	MDA-MB-435 human breast cancer cell, DU 145 human prostate cancer cell, CAL 27 human squamous cancer cell, and HCT 116 human colorectal cancer cell	(Rao et al., 2016a)
	CCM derived from HeLa cancer cell	Two-photon excited (TPE)-NIR nanoprobe	HeLa human cervical cancer cell	(Lv et al., 2018)
	CCM derived from MCF-7 cancer cell	PLGA nanoparticle-ICG	MCF-7 human breast cancer cell	(Chen et al., 2016b)
	RBM	RBM nanoparticle-DOX	HeLa human cervical cancer cell	(Xiao et al., 2019)

## Chemotherapy

Chemotherapy is a class of traditional cancer treatments with broad clinical implementation. Classic chemotherapy can interfere with cell proliferation to achieve cancer treatment. Systemic toxicity and low bioavailability have always been disadvantages that limit further application of chemotherapy. Furthermore, many chemotherapeutic drugs are hydrophobic, which can lead to poor absorption and bioavailability. New chemotherapeutics and existing chemotherapeutics with new formulations have recently been developed to address these limitations (Qi et al., 2017; Guo et al., 2018). Application of nanomaterials as chemotherapeutic DDSs has also been studied frequently. Therapeutic efficacy has been enhanced using nanoparticle-based DDSs. Various CMs have been coated onto nanoparticle surfaces in order to improve the biocompatibility, targeting ability, and circulation time of nanoparticles loaded with chemotherapeutic drugs.

CCM is commonly coated on nanoparticles to endow them with prolonged blood circulation, effective immune escape, and homologous targeting ability. Xu et al. prepared CCM-coated NPs by coating poly(lactide-co-glycolide)-doxorubicin (PLGA-DOX) NPs with HepG2 cell-derived CCM. The resulting CCM-coated nanoparticle size and zeta potential were approximately 100 nm and −29.49 mV, respectively. Due to the tumor specificity derived from homologous binding to CCM molecules, these biomimetic nanoparticles significantly enhanced the cellular endocytosis of DOX toward HepG2 cells when compared to nanoparticles without CCM *in vitro*. Compared to free DOX, the CCM-coated NPs exhibited enhanced antitumor efficacy and reduced system toxicity in HepG2 xenograft mouse models. They benefited from prolonged circulation, immune evasion, and enhanced DOX accumulation at the tumor site (Xu et al., 2019). Tian et al. produced polymer NPs that were co-loaded with hemoglobin (Hb) and DOX and camouflaged with CCM. The resulting nanoparticles exhibited high tumor-targeting capacities. In addition, the nanoparticles could suppress the expression of a series of genes such as hypoxia-inducible factor-1α to abate the exocytosis of DOX, a property that may lead to safe, efficient chemotherapy (Tian et al., 2017).

**Figure 1:**
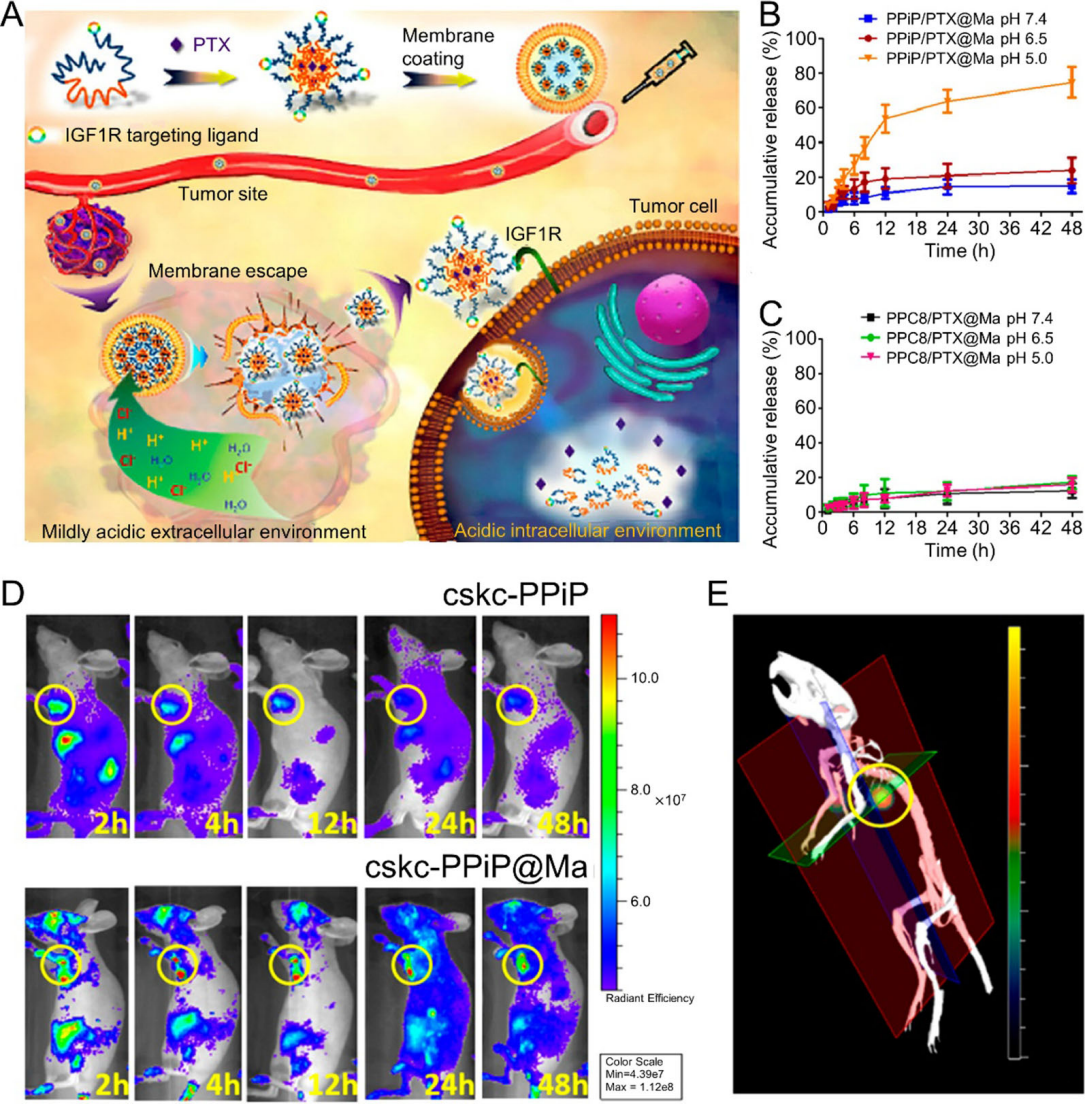
**(A)** Schematic illustrations of membrane-coated nanoparticle synthesis, membrane escape, and drug-release mechanisms. Cumulative drug-release profile of **(B)** PPiP/PTX@Ma and **(C)** PPC8/PTX@Ma in various pH environments. **(D)** *In Vivo* imaging system images of mice after injection of near-infrared probe-loaded cskc-PPiP and cskc-PPiP@Ma at different times. **(E)** 3D reconstruction of the 48 h fluorescence signal of a cskc-PPiP@Ma group. Reproduced with permission from (Zhang et al., 2018). Copyright @ American Chemical Society.

Blood cells include erythrocytes, leukocytes, and platelets and also serve as possible membrane vehicles for CM-coated NPs. Several studies consider blood cell membrane-coated NPs for cancer chemotherapy. Zhang et al. reported RBC membrane (RBM)-coated, DOX-loaded poly(lactic acid) (PLA) NPs. In this study, the authors compared two strategies (physical encapsulation and chemical conjugation) for loading DOX into the PLA NPs. For physical encapsulation, DOX was loaded into the PLA NPs *via* nanoprecipitation. In chemical conjugation, ring-opening polymerization was performed to produce a DOX-PLA polymer conjugate. The resulting DOX-PLA polymer conjugate was added to an aqueous phase to produce nanoparticles. The chemical conjugation strategy led to higher drug loading and more sustained drug release. Furthermore, RBM-coated DOX-loaded NPs exhibited higher toxicity toward acute myeloid leukemia cells than free DOX (Aryal et al., 2013). WBC membranes were shown to act as camouflaged surfaces that help to evade opsonization and RES clearance, as well as inflamed site-targeting navigators. This characteristic may endow WBC membrane-coated NPs with the ability to target some tumors (Fang et al., 2018). Thus, numerous WBC membrane-coated NPs have been developed for cancer chemotherapy, including monocyte cell membrane-coated PLGA NPs (Krishnamurthy et al., 2016), macrophage membrane-coated NPs (Zhang et al., 2018), platelet membrane (PM)-coated core-shell nanovehicles (Hu et al., 2015), and composite CM (derived from leukocytes and tumor cells)-camouflaged liposomal NPs (He et al., 2018). For instance, it was reported that macrophage-membrane-coated NPs could perform step-by-step release of Paclitaxel (PTX) in response to the tumor microenvironment, resulting in tumor-targeting chemotherapy. Macrophage membranes with membrane molecules that exhibit inflammatory tumor-homing abilities were coated onto the nanoparticles. After injection, the macrophage membrane-coated nanocarriers could evade immune clearance and achieve tumor targeting. In an extracellular microenvironment, the nanoparticles could discharge from the outer membrane and further be taken up by cancer cells with help from the targeting peptide on the nanoparticle surface. Then, PTX could be released from the nanoparticles in response to the acidic environment of the endosome. This macrophage-membrane-coated nanoparticle exhibited potent antitumor efficacy (**Figure 1**) (Zhang et al., 2018).

Use of CM-coated NPs to co-deliver chemotherapy drugs and photothermal agents has been studied for cooperative cancer therapy. Ding et al. exploited CCM to enhance the tumor-targeting ability of anticancer drug-loaded mesoporous silica nanoparticles (MSNs). MSNs were co-loaded with indocyanine green (ICG) and DOX, which are a near-infrared photothermal agent and a chemotherapy drug, respectively. CCM was coated onto drug-loaded MSNs to produce CCM-coated MSNs. The CCMs reduced drug leakage during delivery and accumulated at the tumor site efficiently. The photothermal effect of ICG led to CCM fusion and thus accelerated DOX release. This type of CCM-coated MSN could achieve synergistic treatment *via* chemotherapy and photothermal therapy (Ding et al., 2019). Similarly, Zhai et al. developed a cytotoxic T lymphocyte (CTL)-inspired nanovesicle (MPV) with a RBC membrane-derived shell. The CM-coated MPV possessed a gelatin nanogel core, which was co-loaded with methylene blue (MB) and cisplatin (Pt). The CM-coated MPV could achieve deep tumor penetration and produce hyperthermia when stimulated *via* laser irradiation. In addition, photoacoustic imaging (PAI) could be employed to monitor MPV accumulation after injection. Local irradiation could then be used at the time of maximum accumulation at tumor sites. The combination of localized hyperthermia and chemotherapy led by CM-coated MPV inhibited tumor progression while maintaining low systemic toxicity (Zhai et al., 2018). RBM-decorated hollow mesoporous Prussian blue (PB) NPs were also employed for cooperative cancer therapy. These nanoparticles also encapsulated a large quantity of DOX. The authors found that this type of nanoparticle exhibited synergistic photothermal-chemotherapeutic anticancer properties with low toxicity and high efficacy (Chen et al., 2017a).

## Immunotherapy

Cancer immunotherapy can inhibit tumor progression by stimulating immune responses (Li et al., 2018c). In 1986, recombinant interferon-α (IFN-α) became the first immunotherapeutic agent marketed for hairy cell leukemia. After 6 years, recombinant interleukin-2 (IL-2) was also proven effective for metastatic renal cancer by the US Food and Drug Administration (FDA) (Ribas and Wolchok, 2018). Unfortunately, the short half-life of IL-2 can result in serious adverse effects such as vascular leak syndrome. Recent cancer immunotherapy strategies have focused on inducing specific antitumor immune responses. Sipuleucel-T (an autologous active cellular immunotherapy) has been used clinically since 2010. Checkpoint blockade cancer immunotherapies such as cytotoxic T lymphocyte antigen 4 antibody (anti-CTLA-4) and programmed cell death 1 antibody (anti-PD-1) were also demonstrated for clinical applications (Riley et al., 2019). Even though substantial advances have been achieved in cancer immunotherapy, exploring a preventive or therapeutic agent that controls modulation of the immune system, low systemic toxicity, and high antitumor efficiency remains a challenge in cancer immunotherapy applications. This is because such therapeutic agents exhibit serious adverse effects such as nonspecific inflammation and autoimmunity (Qian et al., 2018; Riley et al., 2019). Several strategies have been used to improve therapeutic efficacy and reduce side effects in order to manage cancer immunotherapy in a more controlled manner. Nanoparticle-based DDS can protect immune-related components during circulation and deliver TAAs and immune-modulating agents efficiently. Furthermore, some types of nanoparticle-based DDSs can achieve controlled drug release in response to stimuli like pH in order to harness immunotherapy and reduce systemic toxicity (Li et al., 2018c; Qian et al., 2018). A range of CMs can be used on nanoparticle surfaces to enhance the delivery efficacies of antigens and immune-modulating molecules. In the study reported by Lang et al., platelet and WBC membranes were blended and then coated onto immunomagnetic beads (IMBs). The resulting nanoparticles (HM-IMBs) were modified with anti-epithelial cell adhesion molecules (anti-EpCAMs). The PLT-WBC hybrid membranes could enhance tumor cell binding ability and reduce the homologous leukocyte interaction of the resulting nanoparticles. This can be employed to isolate circulating tumor cells efficiently. Upon testing spiked blood samples, it was found the HM-IMBs exhibited a cell separation efficiency of 91.77%, which compares favorably to 66.68% for IMBs. The cell purity of the HM-IMBs was 96.98%, which is much higher than that of the IMBs (66.53%). HM-IMBs also proved effective in detection of PIK3CA gene mutations (Rao et al., 2018). Similarly, neutrophil-membrane-coated PLGA NPs (NM-NPs) were also developed. The authors found that NM-NPs exhibited enhanced circulating tumor cell (CTC)-capture efficiency *in vivo*. Carfilzomib-loaded NM-NPs could deplete CTCs during circulation and prevent early metastasis (Kang et al., 2017).

**Figure 2:**
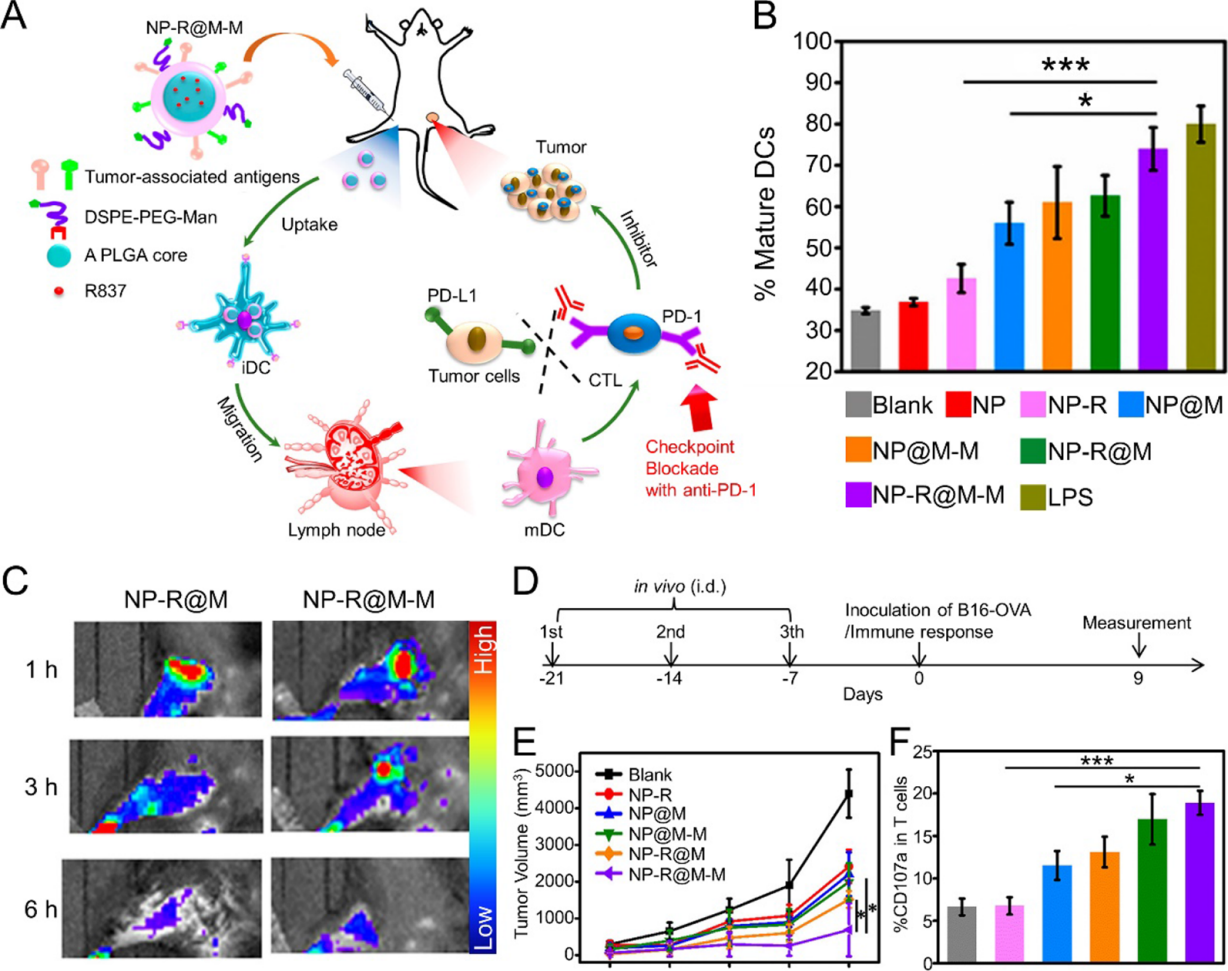
**(A)** Schematic illustration to demonstrate the structures of CCM-coated, R837-loaded, mannose-modified PLGA nanoparticles (NP-R@M-M) and their immune-stimulant functions as a nanovaccine. **(B)** *In vitro* DC activation by various nanovaccine formulations. **(C)** *In vivo* fluorescence images of mouse hind legs after intradermal injection of fluorescent-labeled NP-R@M or NP-R@M-M at three different times. **(D)** Schematic illustration of a tumor challenge experimental design. **(E)** B16-OVA tumor volume curves after pretreatment with various nanovaccine formulations (n ≥ 5). **(F)** Percentages of CD107a+ cells among all T cells. (***P < 0.001, *P < 0.05). Reproduced with permission from (Yang et al., 2018). Copyright @ American Chemical Society.

CCM not only acts on the nanoparticle surface to enhance cargo delivery efficacy but also can be the source of multiple tumor-specific antigens, which are the components of antitumor vaccines. Yang et al. developed CCM-coated PLGA NPs that were loaded with imiquimod (R837). The resulting CCM-coated nanoparticles were further modified with mannose moiety. This nanovaccine exhibited improved uptake by antigen present cells (APCs), which can induce a potent antitumor immune response. When acting as a therapeutic vaccine, the nanovaccine exhibited efficient therapeutic efficacy when combined with checkpoint-blockade therapy (**Figure 2**) (Yang et al., 2018). Furthermore, Manolova et al. found that the nanoparticle size could influence *in vivo* particle migration (Manolova et al., 2008). Li et al. prepared immunoadjuvant-loaded multiantigenic NPs (MANPs/R837) with various diameters. Smaller CCM-coated PLGA NPs exhibited more efficient delivery of antigens and R837 to APCs in draining lymph nodes (LNs) to induce a stronger antitumor immune response. When combined with checkpoint blockade therapy such as the anti-PD1 strategy, MANPs/R837 (especially smaller MANPs/R837) exhibited enhanced tumor progression inhibition (Li et al., 2019).

Synergistic treatment that combines immunotherapy with photothermal therapy or chemotherapy *via* nanoparticle-based DDSs has developed rapidly. Antitumor immune response can be induced *via* photothermal therapy, which can generate TAAs near ablated tumor cells. When photothermal therapy was combined with immunotherapy *via* nanoparticle-based DDSs, potent vaccine-like behavior by therapeutic agents could elicit the elimination of residual and metastatic tumor cells (Chen et al., 2016a). For instance, Liang et al. reported a biomimetic black phosphorus quantum dot (BPQD) formulation that could induce tumor ablation *via* near-infrared (NIR) laser irradiation to elicit an antitumor immune response that further inhibited tumor progression, metastasis, and rechallenge. RBMs were coated onto BPQDs (BPQD-RMNVs) to prolong circulation time and promote tumor accumulation. Moreover, the combination of BPQD-RMNVs and the PD-1 antibody could induce an enhanced antitumor immune response to eliminate cancer cells (Liang et al., 2019). In another study, the authors obtained CCMs *via* surgical removal of tumors. The resulting CCMs were then coated onto BPQDs to get BPQD-CCNVs. BPQD-CCNVs, granulocyte-macrophage colony-stimulating factor (GM-CSF), and lipopolysaccharide (LPS) were loaded into a thermosensitive hydrogel (Gel-BPQD-CCNVs). Dendritic cells (DCs) could be recruited by the GM-CSF released from Gel-BPQD-CCNVs to uptake TAAs. NIR irradiation and LPS could then induce DC maturation. The mature DCs traveled through lymphatic capillaries to LNs to induce a potent antitumor immune response. This synergistic treatment strategy could be combined with checkpoint blockade treatment to improve the antitumor efficacies of tumor-specific CD 8+ T cells (Ye et al., 2019). Similarly, chemoimmunotherapy was proven efficient for cancer treatment. In chemoimmunotherapy, low doses of chemotherapeutic agents can induce immunogenic cell death (ICD) of tumor cells to release TAAs, thus avoiding severe side effects. Immunomodulatory agents can enhance antigen presentation and induce APC and cytotoxic CD8+ T cell maturation. Thus, use of nanoparticle-based DDSs to perform a combination of chemotherapy and immunotherapy was studied and proven efficient (Calleja et al., 2017; Feng et al., 2019). It was reported that a dual pH-responsive multifunctional DDS based on poly(L-histidine) and hyaluronic acid was designed for the delivery of resiquimod (R848, a TLR7/8 agonist) and DOX to achieve synergistic effects of immunotherapy and chemotherapy against breast cancer (Liu et al., 2018). Furthermore, excellent treatment effects might be achieved when CMs are employed to improve the delivery efficacies of synergistic therapeutic nano-agents.

## Phototherapy

Phototherapy is an effective, noninvasive cancer treatment strategy (Chen et al., 2017b). Phototherapy can be initiated *via* laser irradiation to induce selective, localized therapeutic effects. Photothermal therapy (PTT) and photodynamic therapy (PDT) are the two major categories of phototherapy (Vijayan et al., 2018).

### Photothermal Therapy

PTT uses heat ablation generated *via* light-absorbing agents to execute a new, minimally invasive cancer treatment strategy with low systemic toxicity (Chen et al., 2016a). With the development of nanotechnology, it was found that nanoparticle-based DDS could improve therapeutic agent tumor accumulation and the bioavailabilities of water-insoluble cargos. Thus, a large range of nanoparticle-based DDSs have been employed to enhance the delivery efficacies of the light-absorbing agents needed for PTT. CM-coated NPs were recently employed to further improve PTT agent delivery and enhance the therapeutic efficacy of PTT. Meng et al. developed a macrophage membrane-coated magnetic iron oxide NP (Fe_3_O_4_@MM NP) that exhibited excellent biocompatibility, prolonged circulation time, tumor-targeting ability, and effective PTT for breast cancer *in vivo* (Meng et al., 2018). RBC-cancer cell hybrid membranes can also be employed to camouflage melanin NPs, producing Melanin@RBC-M in order to improve therapeutic PTT efficacy. RBMs can prolong nanoparticle circulation times and CCMs can endow nanoparticles with tumor-targeting abilities. The authors studied the delivery efficacy of Melanin@RBC-M formulations with various RBM-to-CCM ratios. The *in vivo* biodistribution and therapeutic efficiency were investigated after intravenous injection of various therapeutic formulations into MCF-7 tumor-bearing athymic nude mice. Melanin@RBC-M with a 1:1 membrane protein weight ratio of RBMs to CCMs exhibited better delivery efficacy and a more potent PTT effect than formulations with other membrane protein weight ratios. Moreover, the authors found that Melanin@RBC-M exhibited an enhanced photoacoustic signal when the nanoparticle size increased from 64 to 148 nm. The photoacoustic amplitude increased linearly with the nanoparticle concentration in the 680 to 800 nm excitation wavelength range. This phenomenon could be employed to quantify Melanin@RBC-M *in vivo* (Jiang et al., 2019).

In addition, PTT can be used therapeutically in combination with chemotherapy or immunotherapy. Liu et al. used hyaluronic acid (HA)-decorated, RBM-camouflaged PB NPs to carry gamabufotalin (CS-6). The study showed that RBM can prolong the circulation time to about 10 h and enhance immune evasion ability. This nanotherapeutic agent could accumulate at tumor sites efficiently because of the HA. Meanwhile, CS-6 exhibited potent antitumor efficacy by inhibiting the expression of HSP70, which can weaken the PTT effect. The resulting nanoparticles exhibited synergistic photothermal-chemotherapy leading to potent *in vivo* antitumor efficacy (Liu et al., 2019). In another study, RGD peptide [c(RGDyC)]-modified platelet vesicles were employed to co-load melanin nanoparticles (MNPs) and DOX to achieve chemo-photothermal therapy for drug-resistant tumors (Jing et al., 2018). In addition, numerous CM-coated NPs have been developed for cancer photothermal-chemotherapy including RBC-melanoma cell hybrid membrane-coated, DOX-loaded hollow copper sulfide nanoparticles (Wang et al., 2018); RBC-camouflaged, PTX-loaded polymeric NPs (Su et al., 2016); RBC-mimetic hollow mesoporous PB NPs (Chen et al., 2017a); and RBC-coated, PTX-loaded gold nanocages (Zhu et al., 2018). Furthermore, synergistic treatment using PTT and immunotherapy was investigated. Liang et al. prepared RBM-coated BPQD (BPQD-RMNV) for combined cancer photothermal- and immunotherapy. Cancer progression and metastasis were substantially delayed *via* improved infiltration of CD8+ T cells into the tumor site when the BPQD-RMNV-mediated treatment was combined with checkpoint blocked therapy (Liang et al., 2019).

### Photodynamic Therapy

PDT can generate singlet oxygen to kill cancer cells when a photosensitizer (PS) is excited by a specific wavelength of light (Feng et al., 2017). RBM (Ding et al., 2015), PM (Xu et al., 2018), stem-cell membrane (Gao et al., 2016), and CCM (Qiu et al., 2018)-coated NP delivery systems were employed to carry PDT agents for cancer treatment. Ding and his colleagues developed an RBM-camouflaged upconversion NP (UCNP). The inner cores were loaded with merocyanine 540 (MC540) and the RBM surface was decorated with targeting moieties including folate (FA) and the triphenylphosphonium (TPP) cation. This formulation could generate ^1^O_2_ under 980 nm irradiation. Meanwhile, the resulting RBM-coated nanovectors could improve singlet oxygen infiltration relative to PDT agents with other surface coatings because of the unique nature of RBC as an oxygen (O_2_) carrier. The combination of the RBM coating and targeting ability significantly enhanced the PDT therapeutic efficiency (Ding et al., 2015). In another study, a PM-coated, verteporfin-loaded photodynamic NP (NP-Ver@P) was developed. P-selectin on a platelet surface could specifically bind with the CD44 receptor that is heavily expressed on cancer cell surfaces. Thus, PM could endow the nanoparticle with long circulation times, good targeting ability, and a higher tumor uptake rate than RBM. Under 680–730 nm solar irradiation, NP-Ver@P exhibited a therapeutic effect against tumors without damaging skin at tumor sites (Xu et al., 2018). CCM-coated NPs have attracted significant attention due to their homologous targeting capabilities. Yang reported coating CCM onto chlorins e6 (Ce6)-loaded silica NPs (CM/SLN/Ce6). CM/SLN/Ce6 exhibited excellent stability in physiological conditions and homologous targeting ability. The aforementioned features make CM/SLN/Ce6 a promising cancer-targeting PDT platform (Yang et al., 2019).

**Figure 3:**
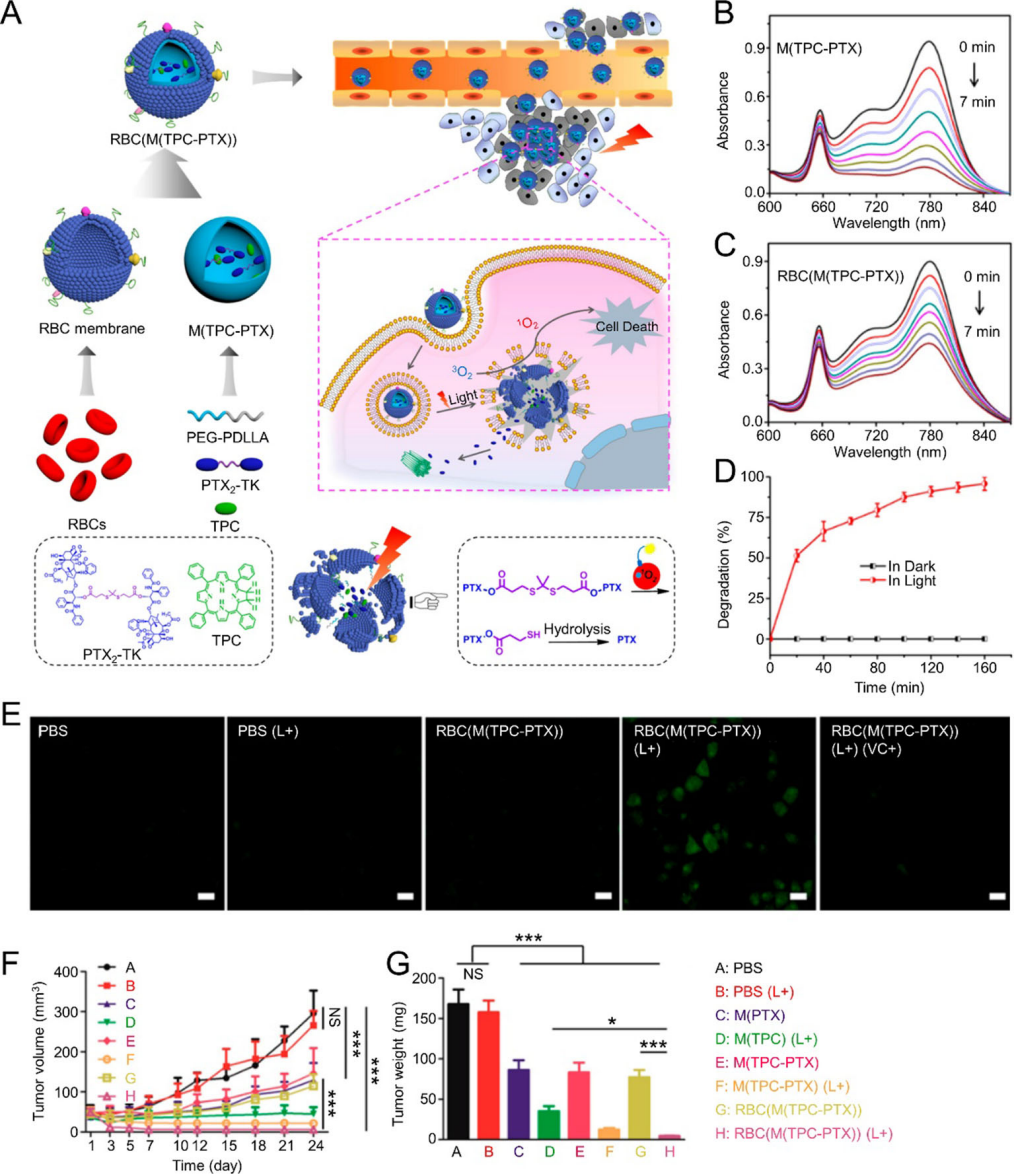
**(A)** Schematic illustration of RBC(M(TPC-PTX)) light-triggered, on-demand drug release for a combination of PDT and chemotherapy. Time-dependent UV absorption spectra of ICG in **(B)** M(TPC-PTX) and **(C)** RBC(M(TPC-PTX)) solutions under 638 nm irradiation (100 mW/cm^2^) for 7 min. **(D)** Degradation of PTX_2_-TK in RBC(M(TPC-PTX)) under 638 nm irradiation (100 mW/cm^2^) over time. **(E)** Generation of intracellular ROS in HeLa cells incubated with various therapeutic formulations. Scale bar = 20 μm. **(F)** Tumor volume curves after treatment with various therapeutic strategies (n = 6). **(G)** Quantitative analysis of tumor weights among various groups. (***P < 0.001, *P < 0.05). Reproduced with permission from (Pei et al., 2018). Copyright @ American Chemical Society.

Cooperative therapy that combines PDT with other cancer treatment strategies has been studied widely. Pei et al. reported RBM-coated NPs (RBC(M(TPC-PTX))) for PDT combined with chemotherapy. Reactive oxygen species (ROS)-responsive PTX dimer (PTX_2_-TK) and 5,10,15,20-tetraphenylchlorin (a type of photosensitizer, TPC) were used to compose the inner core. Light could cause TPC to generate ROS for PDT. The resulting ROS could also elicit PTX_2_-TK cleavage, releasing PTX for chemotherapy. In this platform, PDT and chemotherapy were integrated to achieve high drug loading capabilities and light-induced drug release. Synergetic treatment with PDT and chemotherapy improved antitumor efficacy and on-demand PTX release reduced systemic toxicity (**Figure 3**) (Pei et al., 2018). In another study, a CCM-coated porphyrin metal-organic framework (MOF) was used to load glucose oxidase (GO_x_) and catalase. After accumulating at tumor sites efficiently, the resulting nanoparticles could enhance singlet oxygen production and intracellular glucose decomposition by catalyzing endogenous hydrogen peroxide (H_2_O_2_) to produce O_2_. Furthermore, this O_2_ could promote singlet oxygen production. Induced cooperative therapy using PDT and starvation treatment could inhibit tumor progression efficiently (Li et al., 2017). Furthermore, MR/NIR fluorescence dual-modal imaging could be combined with PDT *via* a CCM-coated nanoparticle delivery system. In a study reported by Li et al., Ce6-loaded magnetic nanobeads were camouflaged using CCM to get SSAP-Ce6@CCM, which exhibited excellent PDT efficacy and MR/NIR fluorescence imaging ability under 670 nm laser irradiation. SSAP-Ce6@CCM might be a promising theranostic platform for tumor treatment (Li et al., 2018a). Li et al. reported a cancer-associated fibroblast cell membrane-coated polymer NP (AF-SPN). AF-SPN demonstrated homologous targeting ability derived from the activated fibroblast cell membrane and used it to target cancer-associated fibroblasts. The AF-SPN core structure is made from poly(cyclopentadithiophene-alt-benzothiadiazole), which is an NIR-absorbing, semiconducting polymer. Thus, AF-SPN can accumulate at tumor sites to induce enhanced NIR fluorescence and PTT and PDT effects for cancer treatment (Li et al., 2018b).

## *In-Vivo *Imaging

In addition to acting as therapeutic drug delivery systems, CM-based NPs are also employed in biomedical imaging applications such as magnetic resonance imaging (MRI), computed tomography (CT), and fluorescence imaging.

Fe_3_O_4_ NPs are a type of novel functional material with low systemic toxicity, high stability, good biocompatibility, and the ability to act as magnetic resonance imaging (MRI) contrast agents (Ren et al., 2016). The delivery efficacies of Fe_3_O_4_ NPs improve when combined with a CM-based membrane. Rao prepared magnetic Fe_3_O_4_ NPs camouflaged with RBM (Fe_3_O_4_@RBC NP). RBM clearly reduced the Fe_3_O_4_ NP RES uptake. Fe_3_O_4_@RBC NPs exhibited excellent potential for MRI and drug delivery applications (Rao et al., 2016b). In addition to MRI applications, superparamagnetic iron oxide NPs (SPIO NPs) were also used for magnetic hyperthermia therapy by their photothermal conversion abilities. Lai et al. prepared stem cell membrane (STM)-coated SPIO NPs for tumor theranostic applications. STM-SPIO NPs acted as potential MRI agents and exhibited excellent magnetization (65.9 emu g^-1^) and dose-dependent T2-weighted imaging contrast (R2 = 653.3 s^-1^ mM^-1^) *in vitro*. Furthermore, STM-SPIO NPs also exhibited magnetic hyperthermia capabilities for cancer treatment (Lai et al., 2015).

Radiolabeled nanocarriers derived from CMs have been studied frequently for non-invasive imaging. Radiolabeled exosome mimetics (EMs) made from RBCs have been used for *in vivo* imaging. RBC-EMs were labeled with technetium-99m (^99m^Tc- RBC-EMs) and exhibited nearly 100% radiochemical purity until 2 h had passed. Furthermore, ^99m^Tc- RBC-Ems exhibited higher liver and spleen uptakes, but no thyroid uptake, unlike free ^99m^Tc (Gangadaran et al., 2018). In another study, Yu et al. developed ^89^Zr-labeled multicompartment membrane-derived liposomes (MCLs). MCLs derived from CCMs were loaded with tetrakis(4-carboxyphenyl) porphyrin. The resulting ^89^Zr-Df-MCLs were used for non-invasive quantitative tracing *via* positron emission tomography (PET) imaging and PDT *in vivo*. ^89^Zr-Df-MCLs demonstrated good radiochemical stability, tumor-targeting ability, and long-term, effective PDT, as well as low systemic toxicity. Specifically, ^89^Zr-Df-MCLs achieved rapid, highly sensitive LN localization (Yu et al., 2018).

**Figure 4:**
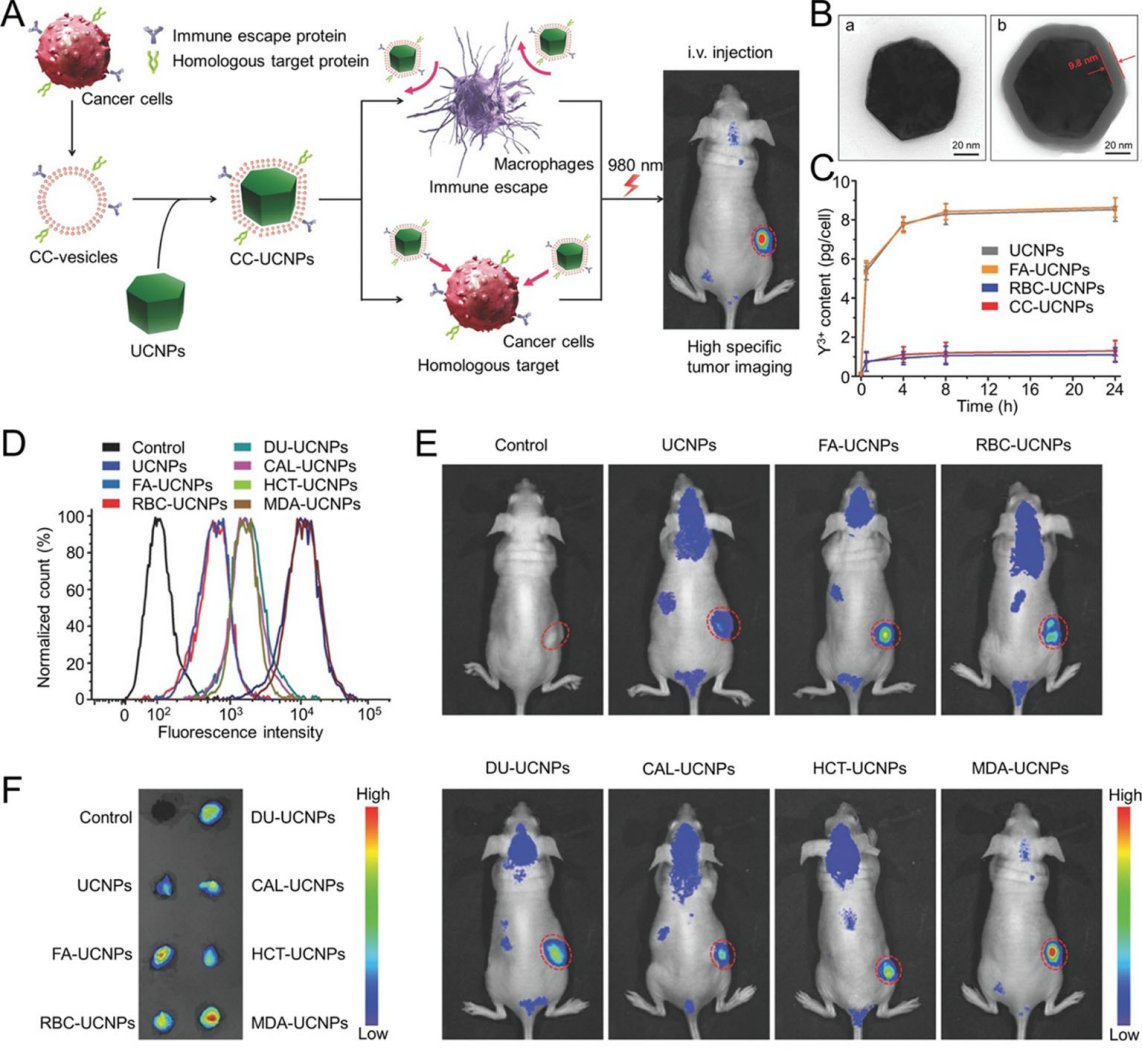
**(A)** Schematic illustration of CC‐UCNP preparation and application. **(B)** TEM images of (a) UCNPs and (b) CC‐UCNPs. **(C)** Quantitative analysis of various nanoparticle uptakes at various times. **(D)** Flow cytometry analysis of MDA‐MB‐435 cells after incubation with various Cy5‐labeled nanoparticles. **(E)** *In vivo* upconversion luminescence (UCL) images of MDA‐MB‐435-bearing mice 24 h after intravenous injection of various nano-formulations (tumor sites are indicated by red circles). **(F)** *Ex vivo* UCL images of tumors 24 h after injection. Reproduced with permission from (Rao et al., 2016a).

Fluorescence imaging is one of the most efficient cancer imaging strategies used in biological studies and clinical applications (Rao et al., 2016a). Rao and his colleagues prepared a CCM-cloaked UCNP (CC-UCNP) that exhibited prolonged blood circulation, immune escape ability, and homologous targeting ability. CC-UCNPs could convert NIR fluorescence into visible light and be used for *in vivo* tumor imaging. They also exhibited potential for tumor diagnosis and treatment (**Figure 4**) (Rao et al., 2016a). Biomimetic fluorescent nanoprobes were developed in another study. These could convert NIR radiation (λ_max_ ≈ 720 nm) into 800 nm light. The nanoprobes exhibited ideal NIR-incoming-NIR-outgoing fluorescence features. The CCM surface imparted the nanoprobes with excellent biocompatibilities and homologous targeting abilities (Lv et al., 2018). Fluorescence imaging could be combined with other treatment strategies using the CM-based delivery system. Chen et al. reported a CCM-camouflaged, ICG-loaded NP as a theranostic nanoplatform (ICNPs). The ICNPs exhibited tumor-targeting abilities derived from the CCM, fluorescence and PAI, and PTT. Benefiting from fluorescence-photoacoustic dual imaging and PTT, ICNPs could achieve real-time tumor monitoring with high spatial resolution and effective tumor treatment (Chen et al., 2016b). In addition to ICG, PB (Xiao et al., 2019), and Ce6 (Li et al., 2018a) were tested in combination with other agents using CM-based nanoplatforms in order to support a theranostic strategy. For instance, Xiao et al. developed RBM-coated, DOX-loaded PB NPs that were modified with FA. The resulting nanoparticles could achieve chemo-photothermal therapy alongside tumor fluorescence and PAI (Xiao et al., 2019).

## Conclusions

CM-based nanoparticle delivery systems have been explored in order to improve on the limitations of traditional nanomedicines. These systems exhibit excellent potential in cancer theranostic applications including chemotherapy, immunotherapy, phototherapy, and *in vivo* imaging. Various CM coatings have attracted significant attention due to the natural features derived from their source cells. CM coatings have been derived from various cells, including RBCs, macrophages, monocytes, neutrophils, platelets, stem cells, and cancer cells. A top-down strategy was used to develop the CM-coating nanoplatform, in which the natural functions of existing cells can be directly transferred to make the ultimate nanomedicine (Narain et al., 2017). RBM can endow RBM-coated NPs with prolonged circulation times, immune evasion, and reduced RES uptake (Gao et al., 2017). WBC membrane-coated NPs exhibit macrophage internalization inhibition because of the WBC membrane (Zhang et al., 2018). CCM-coated NPs have been proven to possess tumor-targeting abilities for drug delivery and vaccine-like functions (Fang et al., 2014; Ding et al., 2019).

Despite current progress in the field of CM-based nanoplatforms for cancer diagnosis and treatment, many challenges remain before they can be used in clinical applications. First, complex, inefficient CM-based nanomedicine preparation processes restrict their development. Coating techniques should be reformed for higher throughputs to satisfy the needs of future clinical applications. In addition, some of the specific functional proteins and structural units in CMs remain uncertain. For example, there are various proteins on the surfaces of CCM-based NPs. Only a few of them act as cancer-specific antigens for cancer immunotherapy. Others are common in human cells. The ability to identify these cancer-specific antigens and remove unwanted antigens or even enrich the expression of cancer-specific antigens through transgenic technology might allow CCM-based NPs to provide better therapeutic effects. Some types of cells such as fibroblasts, which have been used in nanoparticle engineering, have proven difficult to amplify and apply practically in clinical settings. Finally, cancer cell heterogeneity can be a significant factor behind insufficient cancer treatment of CCM-based NPs. Thus, CCMs derived from autologous tumors may serve to provide specific CCM-based NPs for cancer treatment.

In conclusion, research on CM-based nanoplatforms for cancer diagnosis and treatment is still in its infancy. Numerous challenges must be overcome before the translation from bench to bedside. More innovative, efficacious CM-based nanoplatform strategies will be developed to support cancer treatments that benefit human health.

## Author Contributions

SL and JL produced the first draft. MS and JW revised the manuscript. CW and YS proposed the outline of the article and revised the draft before submission. In addition, all authors provided final approval of the manuscript.

## Funding

This work was financially supported by the National Natural Science Foundation of China (Grant Nos. 5177030177).

## Conflict of Interest

The authors declare that the research was conducted in the absence of any commercial or financial relationships that could be construed as a potential conflict of interest.
